# A Reply to the Editor and Publisher of the Dental Cosmos

**Published:** 1864-08

**Authors:** B. Wood

**Affiliations:** Albany


### DENTAL REGISTER OF THE WEST.

Vol. ~~XIX.]~~ XVIII AUGUST, 1864. [No. 8.

### Original Essays and Communications.

### A REPLY TO THE EDITOR AND PUBLISHER OF THE DENTAL COSMOS.

BY B. WOOD, M. D.

May I claim the attention of your readers to some remarks in reply to the communications in the April and May numbers of the "Dental Cosmos," by the Editor and Publisher respectively of that publication, which were calculated, not merely to damage my own interest and reputation, and to mislead those in search of information on a subject of practical moment, but to affix a slur upon gentlemen of the highest standing in the profession. The subject in its bearings is of general concern, whether as relating to the resources of the profession or to its members. I regret that it should have been forced to assume a personal character, but am left no other alternative. The conductors of journals have great influence for good or bad—if they use it to improper ends, especially if in doing so they leave facts and argument and resort to personal assault, they are properly held to personal account.

It is no plea for unfounded representations to pretend that the thing sought to be put down is worthless, but only the more reprehensible, since if that were the fact there would be no occasion for misrepresentation. Neither can foul means to crush an individual be extenuated on an assumption or pretense discreditable to his good name:—it is of vital interest to all to know if the means *are* foul, and, doubtless, he has a right to a statement of his case that the profession may judge; more than this it is a duty he cannot shirk and be true to the common interest: for if such means be practiced in any case on the part of journals, the influence of which is so formidable as against individuals, then every man in the profession stands in jeopardy, and the more in proportion to his real merit and standing, for if exercised against the low and unmeritorious, how much more likely to be, should caprice or malice prompt, in respect to those whom a fair and ingenuous course could not harm.

These reflections will, it is hoped, exonerate from the charge of presumption in asking a somewhat full reply to imputations from influential sources.

An attentive examination of the articles in question discovers no fact or argument against the *material*, and not a single point disproved or met in relation thereto—the whole having a personal drift, with singular unity of purpose to impeach and discredit its inventor, and to slur the fair name of those who had dared, or might dare, to speak in its favor.

All Dr. White has to oppose to "Wood's Plastic Metallic Filling" is comprised in the following: "We do not find any use for Plastic Filling in our practice, because we do not use any thing for plugging teeth but gold, Hill's stopping, Oxichloride of Zinc and Tin;" and the implied objection when saying that amalgam "has not the disadvantage of complicating the manipulations with it by using heat;"—(although it is well known that, as most successfully used, the "manipulations with it" *are* "complicated by using heat.") This is the totality of what he opposes:—1st, He does not find use for it "*because*" he does not use it. And 2d, it is complicated with heat. This is the sole argument. (What he says of "White's Terra Metallic Compound" and other mercurial preparations is nothing to the purpose.)

Dr. S. S. White adduces no argument or fact whatever.

He says "Dr. J. D. WHITE has PRONOUNCED" against it, as if that were conclusive, and contents himself with implied corroboration of Dr. J. D. White's personal imputations.

There is therefore no fact or argument to meet as against the material; and no alternative left but a personal defence. Was it hoped that forcing this attitude might preclude the hearing of a reply on the ground of "personality"?

Let us first in order and detail examine

DR. J. D. WHITE'S EDITORIAL, (APRIL NUMBER COSMOS.)

As the number containing this has been freely circulated among non-subscribers and has probably reached every nook and corner of the profession, (perhaps as an earnest of "the *efforts* to render Dr. Wood justice,") I would like, if not so wide, at least a patient hearing in reply.

The caption of the article—"B. WOOD'S IMPROVED PLASTIC METALLIC FILLING"—*in quotation marks*, implies more than the slur which it attempts, while it betrays the unfortunate temper and animus with which the writer sets out; for the drift of the article purports that this material had been used before, and that HE discovered and used precisely the same thing several years ago.

Dr. White proceeds to say that he had been written to by dentists to know what he "thought of Wood's Plastic Filling," that he has not answered these letters; "but," he adds "the following letter we feel bound to answer, because it contains a moral as well as a practical meaning:"—He then copies a private note addressed by me to him, dated August 12, 1863, enclosing him a sample of Plastic Metallic Filling for trial, and remarking: "You have great influence; you can retard the use of a good thing, (and thereby impair that influence,) or you can extend its benefits." Proceeding to reply to this note (which he "feels bound to answer") he says: "Now, we had tried the plastic filling before receiving the above letter, but did not intend to say anything about it, unless it came up in a way that made it necessary." This note, then, of August 12, 1863, brings up the subject in a "way that makes it necessary" for him, April 1864, to "say something," which, accordingly, he goes on to "say:"—making this note, received ten months previous, the occasion and provocation of his present saying.*

It is note worthy that the editorial avoids mention of the article on the "Disposition made of Communications," which had appeared in the *Dental Register* for March, leaving that to the hands of the publisher, although the allusion to "advertisements" "published gratuitously," and the drift throughout, partake of the nature of a reply thereto. Was not this the real ground of offence which brought the matter up in a "way that made it necessary" to say something? Was not this the occasion which reproduced that private note, so carefully preserved for nearly a year in waiting? At any rate, the discussion involving principles is evaded, but immediately the "letter" assumes an importance of such magnitude that the editor, who has "not time" for reply to any others, "*feels bound* to answer it"—"because" it is suddenly discovered to "contain a moral as well as a practical meaning." Dr. White, under the circumstances, could hardly have misunderstood the meaning, however exceptionably worded—not as a patronizing "solicitation" for his commend-

---

*Perhaps the parading, for stricture, with the most unfavorable construction, of a careless private letter, written at the spur of the moment and sent off, not considering to *whom*, nor caring or remembering what was written, and least of all surmising the possibility of its being paraded in public print, will be justified upon the ground that, in appealing from the decision of the Cosmos in the case of a rejected communication, I had published the grounds of objection. But, in such cases, the publication of communications or of the reason given of their rejection, is optional with the recipients. It is public matter, if either party choose to make it so. When an appeal is made it may become the party making it to state the fact, and he may or may not publish or take issue with the adverse decision, as he sees fit. Hence editors weigh their objections and are less apt to decide capriciously. The objections returned by the publisher with my communication, in behalf of the editor and himself, had been drawn up after consultation and "due consideration," as though in anticipation of publication; and in the next issue the Cosmos leads off by the publication, in part, of a private letter from me, in its Review department, with some strictures thereon. This will suffice for the allusion in the publisher's article to my "reviewing" his "letter."

ation of the Filling, which was never asked, but an appeal to prove it fairly, and not prejudice its introduction without a *trial.* It was an expostulation against what has now become overt, accompanied with a prophecy which would now seem likely to be fulfilled. If it was written in the temper of a forlorn hope, it was at least an honest expression of what was felt at the moment. I do not despise the "influence" of any one, much less that of an editor having, to say the least, the full confidence of one who held almost my entire interest at his will; although I should scorn upon any pressure

"To bend the supple hinges of the knee
That thrift might follow fawning."

Dr. White in his editorial capacity surely has power to retard the use of improvements or to extend their usefulness. What *good thing* originating with his compeers has he not retarded? And has not his influence been sometimes felt in the introduction of things not good? Was not that influence already somewhat "impaired" in consequence!

I may remark that this was the only thing ever addressed to Dr. White on the subject, except a line about a year previous, which I remember quite distinctly being a copy of what was addressed to Dr. Taft the same time (date forgotten) substantially as follows:—"Dear Sir: I take the liberty to enclose you a sample of my Plastic Metallic Filling for trial. Yours truly," &c. (Dr. Taft at once gave it a trial and reported the result to his readers. Several months afterwards "Dr. White had not found time to try it.") This comprises the extent of my correspondence with Dr. J. D. White. It involves the sum total of solicitation for editorial influence. Yet the doctor's editorial would lead to the inference that I had importuned his "influence" until past endurance!

If any apology were necessary for sending him a second sample and urging a trial of it, I may state that although made sensible of his prejudices, yet the favorable effect which the testimony elicted at the American Dental Convention seemed to have in removing those which Dr. S. S. White confessed to have before entertained, in connection with the reflection that the first sample sent was of No. 2, with which some dentists had failed who succeeded with No. 3, prompted me to send a sample of my "*improved No.* 3 Plastic Metallic Filling" (as I suppose it was worded.)

But the doctor had tried it before, &c. He presently says he does not find use for it "because" he does "*not use any thing*" but certain other articles enumerated. How is a thing to be *tried* without using? When and why did he cease its use after having fairly acquired it? for it would be interesting as the first instance known. But he says, further on, of an old mercurial compound that it was used "precisely as Dr. Wood's;" which is conclusive that he does not understand how the latter is used, for the manipulation is quite different and requires peculiar instruments never before known or used. Will the doctor report the number and nature of the cases in which he "*tried*" it, and what instruments he employed?

Dr. White continues to inform his many inquirers that "metals applied by heat are not of recent date;"—(that was before told, and their compositions and qualities, with opinions of the old writers for and against, were given in a communication "accepted by Dr. J. D. White"—and for the publication of which in the Cosmos I am now held as largely indebted;) and that he has had "some experience in that direction." He then adduces a testimonial handed to him "without solicitation, among others of a similar import from eminent medical men" relating to "*his* metallic compound" and his skill in the use thereof. "This metallic compound," Dr. White states, "was used by heat, precisely as Dr. Wood's, and looked as much like it as possible. It was no better than amalgam except that it contained less mercury." Here follows another testimonial to "WHITE'S TERRA METALLIC COMPOUND," which it appears from a former account he discovered "by a series of experiments," &c.—and used under this "scientific name" in 1838–39. Proceeding to confound the two and assuming their identity, he makes out as though my case were a parallel to his own and thereupon undertakes to affix a stigma upon me over his own shoulders.

The doctor has recorded the history of his case, which will serve to test the parallel—(See *Dental News Letter*, October 1854.) It is to be remarked that in all he has said of this compound upon which he now assumes so much, he has never yet divulged the *secret* of its composition, &c., which is the more surprising since by that he could have invalidated a patent subsequently obtained for the same or a similar thing.

But let us trace the history from his record quoting the substance in his own language. His preceptor used a preparation which was applied hot "but which was held from us a secret.... But we as an ambitious student did not wish to be cut off.... We therefore entered upon a series of experiments, aided by an intelligent physician and obtained a similar substance.... We used this substance under the scientific name of *Terra Metallic Compound*, and proposed to use it for such cases *only* as could not be plugged with gold and roots of teeth [?] We used this for five or six of our medical friends, who declared it to be invaluable, and four of the number voluntarily handed us certificates of its merits.... The following is a true copy of one of those flattering testimonials of our *skill* at that time." Here follows a letter dated Philadelphia, 9th March, 1839, commendatory of White's Compound, and concluding by expressing a "high opinion of the skill and dexterity displayed," and subscribed, "A—— D——, M. D. To Mr. White, Surgeon Dentist."

"Now I would ask" continues Dr. White, "would it be surprising if such an array of complimentary certificates would not turn the brain of a young dentist with a goodly share of self-conceit?" But being "advised" to get the endorsement of a Professor of the University of Pa.;—"Judge of the terrible blow we, poor innocent, was [?] doomed to receive." For, the "learned Professor" looking "at the author's name, and then to the purport of the letter," thrust it back, &c., (as detailed in the editorial, but where it is added, "when he became acquainted with the object of the writer,"—not the innocent *bearer!*) with the exclamation "'*Tis a prostitution of the name*," which sent him away "blushing with shame." "So we laid our letters and *compound* away and applied our energies and talents to a different class of studies. We adhered to *pure gold* as a material for plugging teeth" &c. (the italics are the doctor's.) "And.... have long since met this same Professor.... without shame. He has been of great value to us, the others alluded to of absolute injury; he is still one of the brightest lights in the scientific world, but the rest remain in obscurity."—Thus closes the record from the *News Letter*.

Now, it may be remarked that although the Professor was right; (whose chemical knowledge could not but recognize this reputed discovery as an old mercurial compound, and he probably was advised of its having been used for filling teeth, whether from dental authors or intelligent dentists; or, if not, saw at once the impropriety of signing certificates to what related to a different branch, and about which he could know nothing practically;) yet it was no extenuation for flouting his language in the face of those who sought to befriend Dr. White believing him honest, acquainted with the nature of his own business and to have really made a discovery. But it would appear from intimations that they afterwards proved "false friends," not only of no "great value" to him, but of "absolute injury."

Turning to Dr. White's editorial, we now find him giving the names of the parties—thus far elevating them from that obscurity to which they were before consigned. He does more. He now says: "The letters were received from men of education and influence, and one of the gentlemen, who is still living in our city, is of the highest integrity and scientific acquirements." Are we left to conclude that subsequent to the first writing this gentleman had become of "great value" to Dr. White?

But having first thrown *all* of the Professor's rebuke from his own shoulders upon those of "ill-advised friends," the Doctor now lightens their burden to hurl it upon a new class of objects that have stirred his ire.

"Now," continues he, "these good gentlemen, and the worthy Professor, may have been wrong;" (they are all good now, and elevated on a par and in the same category with the Professor;) "but we" (never presumed to be wrong, but always mounting higher,) "left off the use of the compound and applied ourselves" (how many "selves" has the Doctor) "more energetically than ever" (which before must have been *very* energetic) "to the study of the science of medicine, from which we hoped to draw wholesome lessons and knowledge, by which we might become capable of meeting the wants of our patients," (the Doctor's editorials never lose view of *patients*,) "and obtain a better understanding of the principles of physiology, pathology, and the patient's physical well-being, than could be obtained from the dental works then extant, or any mechanical trick in the trade, or secret compound to fill teeth, without regard to the consequences."

Thus, having ransacked all these varied and heterogeneous sources, without regard to consequences, ought not the Doctor's "influence" to be great and his decisions final? Ought he not to shape our literature, sit in judgment, and be the oracle of the profession?

But waiving these questions, let us look into the state of his case in the matter before us, and see what becomes of this high sounding jargon. He still holds on to his "secret," but in zeal to strike down something else, lets out that it was a mercurial compound. Years before that, Darcet's Alloy amalgamated with mercury, had been applied to filling teeth, and was described in "dental works then extant," which had he read them as he professes, he would have known; or by studying the chemical text books he might have learned the effects of mercury on such alloys and so found out his preceptor's secret. But "as an ambitious student," (ignoring the books,) he "entered upon a series of experiments, *aided*

by an intelligent physician" (who doubtless was enough posted in chemistry to direct) "and obtained a *similar substance*:—" (this is related when professor in a dental college, with an air of mystery, not divulging what it was, as that would have lowered the scientific pretensions based upon it.) He takes for it the name of "*White's* Terra Metallic Compound." What next? Does he publish his discovery? or, verily believing it to be new and useful, and desirous of securing remuneration, make it *patent*, for the benefit of science, filing a "true and exact description thereof" in the public archives? Does he submit it for trial and use to his fellow practitioners, rather than appropriate it to his own use as against them? Does he court *their* examination and opinion? Not at all. He uses it, with sounding name, and according to his own confession "without regard to consequences," obtaining "voluntary" certificates, not from competent members of his own profession, but from members of another unaquainted with the wants and requirements. But he was young and "innocent." It is said those who start out in the world without certain fixed principles will rarely find them.

It would seem he was governed by other than principle even in abandoning his "trick in the trade and secret compound." It was "*shame*" first; next policy, for he found a "barrier to success in life" to "baffle the machinations of quackery." "So" he laid them one side. But had he at the time been conscious of the novelty and utility of the thing he would not have slunk away at the first rebuff. Nor, had he offered something novel to the Professor in his own province to judge upon, for professional use, the Professor would not have so turned him off, but if, upon due inspection or trial, found useful, would have cheerfully seconded its introduction to his medical brethren—without regarding this a "prostitution of the name."

So much as to the parallel of the case upon the assumption of which Dr. White hopes to find other lodgement for odium cast.

Dr. *J. D.* White is very severe upon testimonials,—and falls indiscriminately on those who give them. Turning over the pages of the same number of the Cosmos, wherein he

"Showers his wild blows like wintry rain,"

there are found, in the advertising sheet, some thirty testimonials to Dr. *S. S.* White's foil, with perhaps fifty professional names, among them these of Drs. McQuillen, Kingsbury, Atkinson, Kingsley and other eminent dentists—probably Dr. J. D's would have been in the same good but for appearance sake, as editor for Dr. S. S.—"*It is a prostitution!*" And the publisher give *unusual* circulation, to this denouncement of his friends! But have not dentists a right to give their honest impressions, public or private, in regard to any material made use of in the profession, by whomsoever offered, and to commend it, if in their opinion commendable? Do they not owe it to one another for mutual information? Unquestionably. And they may give it as a communication or allow it to go as an advertisement, according to circumstances. It is only in this way new improvements are to be made known in the face of powerful interests or exclusiveness on the side of rival articles already in general use. It is a kind and generous hand, which aids the new comer in its buffettings for existence in a world of prejudices and hostilities.

But while Dr. White has great repugnance to testimonials, in certain cases, yet he does not object to parading them if they relate to himself. This temptation he can not resist. He may turn and rend those who bestow them, but takes care to treasure and display the pearls. If there is any dearth he supplies the void from his own pen. Witness, *passim,* the editorials of the Cosmos.

Nor does he appear to be particular whence they emanate, only so that they come.—I quote the following for a sample —not from "eminent medical men" but from a "grateful patient." It is from an aged lady, published in an editorial by Dr. White in the Cosmos for Oct., 1862, and testifies to the Doctor's performances with Oxichloride of Zinc; in regard to which the Doctor relates that "ragged and some loosened teeth were plastered up with it to look like *whole* teeth"! (The italicising is his own, as well as in what follows.)

"March 16th, 1860.

"Mrs. D., with the enclosed $50, returns Dr. White *very many* thanks for his kind attention—for his *professional skill*, which answers perfectly.

Portico square."

On a former occasion, having met a "barrier to success in life," he informs us that he applied his "energies and talents" differently, and "adhered to *pure gold*," &c.; and later he intimates his abandonment of "any mechanical trick in the trade, or *secret compound* to fill teeth, without regard to the consequences." He assures us he does not believe in "*metallic compounds*, as such;" implying an apprehension of their liability to oxidize in the mouth. But he employs, recommends, and publishes testimonials of "professional skill" in the use of a compound composed of *metallic oxide* itself, with a corrosive liquid, and other questionable ingredients; (of which he says, *Cosmos*, August. 1861, that while he believes "that *where a tooth is strong and firm*, gold is *still* the best, the zinc filling is *invaluable* in many cases," &c.;) and all this palpably "without regard to consequences," not knowing the nature of the compound; for in a later article in regard to it, *Cosmos*, March, 1863, he confesses—"it is said that acid and alkali destroy it; *we know nothing about the chemistry of the subject.*" He is constrained to admit also, at the same time, that "it is a very uncertain and unreliable substance," over which it is "necessary to keep watch that it does not wash or wear away, as there is no certainty how long it will last"—that "while it may remain for two or three years in a tooth, still it *washes* away in many cases in a much shorter time," &c. Yet, before and since, he has applied himself more to introduce and sustain this article than all the decided *improvements* put together.

Dr. White makes large and repeated pretensions to extent and profundity of medical knowledge, which if sifted would show of a type with those already referred to. But thus far became necessary to expose, since he has wielded—not facts and arguments—but the weight of his influence to give force and credence to imputations as baseless as they were designed to be injurious.

ALBANY, June, 1864.

